# Application of Engineered Bacteriophage T7 in the Detection of Bacteria in Food Matrices

**DOI:** 10.3389/fmicb.2021.691003

**Published:** 2021-08-06

**Authors:** Nicharee Wisuthiphaet, Xu Yang, Glenn M. Young, Nitin Nitin

**Affiliations:** ^1^Department of Food Science and Technology, University of California, Davis, Davis, CA, United States; ^2^Department of Nutrition and Food Science, California State Polytechnic University Pomona, Pomona, CA, United States; ^3^Department of Biological and Agricultural Engineering, University of California, Davis, Davis, CA, United States

**Keywords:** engineered bacteriophage, *Escherichia coli*, colorimetric detection, alkaline phosphatase, 5-bromo-4-chloro-3-indolyl phosphate

## Abstract

Detection of pathogens in a food matrix is challenging due to various constraints including complexity and the cost of sample preparation for microbial analysis from food samples, time period for the detection of pathogens, and high cost and specialized resources required for advanced molecular assays. To address some of these key challenges, this study illustrates a simple and rapid colorimetric detection of target bacteria in distinct food matrices, including fresh produce, without prior isolation of bacteria from a food matrix. This approach combines bacteriophage-induced expression of an exogenous enzyme, alkaline phosphatase, the specific colorimetric substrate that generates insoluble color products, and a simple filtration method to localize the generation of colored signal. Using this approach, this study demonstrates the specific detection of inoculated *Escherichia coli* in coconut water and baby spinach leaves. Without isolating bacteria from the selected food matrices and using a food sample size that is representative of industrial samples, the inoculated samples were added to the enrichment broth for a short period (5 h) and incubated with an engineered bacteriophage T7 with a *phoA* gene. The incubation period with the engineered bacteriophage was 30 min for liquid samples and 2 h for fresh produce samples. The samples were then filtered through a 0.2-micron polycarbonate membrane and incubated with a colorimetric substrate, i.e., nitro blue tetrazolium/5-bromo-4-chloro-3-indolyl phosphate (NBT/BCIP). This substrate forms a dark purple precipitate upon interactions with the released enzyme on a filter membrane. This approach successfully detected 10 CFU/ml of *E. coli* in coconut water and 10^2^ CFU/g of *E. coli* on baby spinach leaves with 5 h of enrichment. Success of this approach illustrates potential for detecting target bacteria in food systems using a simple visual assay and/or quantitative colorimetric measurements.

## Introduction

Globally, human illness due to foodborne pathogens is a major cause of morbidity and mortality. Even within the United States, which has advanced sanitation systems, an estimated 48 million people experience foodborne illness each year (Centers for Disease Control and Prevention, 2018). The leading cause of foodborne illnesses has been the contamination of food, such as fresh produce, dairy and meat products with microbial pathogens, for example, among those pathogens of most concern are Shiga toxin-producing *Escherichia coli* (STEC), which are reported as a cause for an estimated 265,000 illnesses and 3,600 hospitalization annually in the United States (Scallan et al., 2011). Therefore, rapid and efficient detection of the microbial contaminants plays an important role in mitigating the risk of food borne outbreaks and improving sanitary controls in the food supply chain. Several approaches for pathogen detection have been deployed in the industry. However, rapid and sensitive detection of bacteria, especially in complex food matrices, continues to be a challenge.

In the food industry, multiple detection methods are used for the food safety assessment process. The conventional culture-dependent detection method is considered as the gold standard for bacterial detection; however, it is both time-consuming and labor-intensive. These limitations can be acute for food industries, such as the fresh produce industry, as products have limited shelf life (Cho and Ku, 2017). Therefore, more advanced rapid detection methods, including nucleic acid-based and immunological-based methods, are currently being employed in the food industry. Polymerase chain reaction (PCR) is a nucleic acid-based technique offering rapid and specific pathogen detection. This approach has high sensitivity enabling simultaneous amplification and quantification of specific nucleic acid sequences (Kralik and Ricchi, 2017). The most commonly used immunological-based method is enzyme-linked immunosorbent assay (ELISA) that can be automated to enable rapid detection of pathogens with reduced labor.

However, these advanced methods also have some drawbacks. PCR has suffered from complicated sample preparation, costly reagents, and technical support requirements. Using nucleic acid-based methods to detect pathogens in food samples can be challenging since foods are highly complex biomolecular matrices with diverse arrays of biomolecules such as proteins, carbohydrates, fats, oil, polyphenolics, and other small molecules. These compounds may act as inhibitors of the enzymatic reactions in PCR, resulting in false-negative results or limit sensitivity of the assay (Jaykus, 2003). The use of PCR in food is also limited by high sample volumes (≥25 ml or g) compared to small amplification volumes (10–50 μl) used for the PCR detection (Stevens and Jaykus, 2014). In the case of ELISA, reduced specificity due to the cross-reactivity of polyclonal antibodies with closely related antigens can be a limitation (Hornbeck, 2015). Therefore, constraints for the detection of foodborne pathogens by ELISA include a large number of background microflora present in food samples. Even though these microbes may not cause any health problems, they can interfere with the selective identification and isolation of target pathogenic bacteria, which are usually found in relatively low numbers. In order to improve the sensitivity of these detection methods, at least 4–5 h of enrichment is still required for both PCR and ELISA assays, which prolongs the overall turnaround time. Despite these enrichment steps, the detection sensitivity of typical RT-PCR assays using the isolation and detection of bacteria from food matrices ranges from 10^2^ to 10^4^ CFU/ml and for ELISA assays ranges from 10^3^ to 10^5^ CFU/ml (Sharma and Mutharasan, 2013). In addition, the standard PCR and ELISA methods provide limited specificity for differentiating live vs. dead microbes (De Boer and Beumer, 1999; Liu et al., 2017).

Considering the above challenges, there is still a significant unmet need for rapid, cost-effective, and easy-to-perform bacterial detection methods that can be applied to detect specific bacteria in the presence of complex food samples. Bacteriophages have become a valuable tool for developing bacterial detection assays due to their ability to recognize and infect specific strains of bacteria with the time of analysis ranging from 15 min to 8 h (Paczesny et al., 2020). For the detection of bacteria in complex food matrices, the common approach is to genetically engineer bacteriophages to express reporter genes upon infecting the target host bacteria. One of the most commonly used reporter genes is bacterial luciferase (*lux*) that generates a bioluminescence signal (Loessner et al., 1997; Kim et al., 2014), for example, the *E. coli* O157:H7-infecting bacteriophage phiV10 was genetically modified to express the *lux* operon and used for the detection of *E. coli* O157:H7. This approach was reported to detect 10 CFU/cm^2^, 13 CFU/ml, and 17 CFU/g of *E. coli* O157:H7 in romaine lettuce, apple juice, and ground beef, respectively, by detecting bioluminescence using a luminometer with 5-h pre-enrichment (Kim et al., 2017). Another commonly used reporter gene is *lacZ* encoding β-galactosidase. Using this approach, *E. coli* infected with T7 bacteriophage with the *lacZ* operon overexpresses β-galactosidase and allowed the detection of 10 CFU/ml of *E. coli* using a colorimetric substrate within 7 h and enabled the detection of 10^2^ CFU/ml of *E. coli* in food samples (Chen et al., 2017a,b). The gene that encodes alkaline phosphatase (*phoA*) is another reporter gene that has been engineered into the bacteriophage T7 genome to induce the overexpression of alkaline phosphatase. Overexpression of alkaline phosphatase gene has been detected using both soluble colorimetric substrate, p-nitrophenyl phosphate (pNPP), which allows for the detection of 10^4^ CFU/ml within 7.5 h and 10^3^ CFU/ml of *E. coli* in 6 h using a chemiluminescent substrate (Alcaine et al., 2015). Enzyme-labeled fluorescence-97 (ELF-97), alkaline phosphatase substrate that gives insoluble fluorescent precipitated products, has been used to detect the engineered bacteriophage-induced alkaline phosphatase activity at the single-cell level using fluorescent imaging and image analysis. This approach was tested in beverage samples and was able to detect 10^2^ CFU/ml of target bacteria within 6 h (Wisuthiphaet et al., 2019). Even though these methods have been proven to detect a low number of the specific bacteria in complex matrices, maintaining both rapidity and simplicity of the procedure can be challenging due to a range of issues related to sample preparation including bacteria isolation steps, instruments required for signal analysis, and distinguishing signal from high background noise in food samples.

The goal of this study was to develop a rapid bacteriophage-based colorimetric bacterial detection that is low-cost, easy-to-perform, and can be applied to detect bacteria in complex food samples without isolation of bacteria from food matrices. Isolation of bacteria from food samples is a time, equipment, and labor-intensive process (Stevens and Jaykus, 2014). The approach evaluated in this study does not use any isolation of bacteria from food samples and thus reduces complexity of the assay. Furthermore, colorimetric detection approach was selected as it provides a simple read out for a visual analysis as well as quantitative measurement using a camera. For colorimetric detection, we evaluated insoluble substrates, nitro blue tetrazolium and 5-bromo-4-chloro-3-indolyl phosphate (NBT/BCIP) and commonly used soluble substrate, pNPP to detect the enzymatic activity. NBT/BCIP yields insoluble dark purple precipitate that is localized at the site of the reaction and can be visualized by the naked eye. After applying this substrate to the bacterial cells with overexpressed alkaline phosphatase, the precipitated product was accumulated inside the cells. After separation of bacterial cells from the combined culture medium and the food samples by filtration, the signal was concentrated and was enabled the detection of visible color change. The efficiency of bacterial detection using NBT/BCIP and engineered bacteriophage T7 was evaluated in coconut water and baby spinach leaves and compared with those of the commonly used soluble substrate, pNPP. Overall, the results of this study demonstrate the potential of visual detection of bacterial contaminants in food samples using engineered bacteriophages without extensive sample preparation steps.

## Materials and Methods

### Bacteriophage and Bacterial Strain

Engineered bacteriophage used in this study, designated bacteriophage T7-ALP, was bacteriophage T7 that has been genetically modified to carry the gene for alkaline phosphatase production, *phoA*. The bacteriophage T7-ALP strain was kindly provided by Dr. Sam Nugen (Alcaine et al., 2015). *Escherichia coli* BL21 (ATCC BAA-1025) obtained from the American type culture collection was used as a host for the bacteriophage T7-ALP. Bacteria were stored in tryptic soy broth (TSB; Sigma-Aldridge, St. Louis, MO, United States) containing 15% (vol/vol) glycerol at −80°C. For short-term storage, the glycerol stock was streaked onto tryptic soy agar (Sigma-Aldridge, St. Louis, MO, United States) plates. After incubation at 37°C for 24 h, the culture plates were stored at 4°C. Overnight culture of *E. coli* BL21 was prepared by inoculation a loop-full of culture on an agar plate in TSB, after 16 h of aerobic incubation at 37°C, bacteria with the concentration of 10^9^ CFU/ml were obtained.

### Sample Preparation and Bacterial Inoculation

Overnight culture of *E. coli* BL 21 was centrifuged at 16100 ×*g* for 1 min. The cell pellet was washed twice and re-suspended in sterile phosphate buffer saline (PBS; Fair Lawn, NJ, United States) at the population of 10^9^ CFU/ml. The serial dilutions were performed using sterile PBS to obtain bacterial concentrations of 10^3^ and 10^2^ CFU/ml.

Sterile TSB was portioned to 10 ml in a 50-ml sterile centrifugal tube. Pasteurized coconut water (Vita coco 100% coconut water) purchased from local grocery store, and 5 ml was mixed with 5 ml of double concentrated TSB in a 50-ml sterile centrifugal tube. *Escherichia coli* BL21 with the final concentration of 10 CFU/ml was inoculated in 10 ml of the liquid media. The fresh produce sample was represented by baby spinach leaves. Store-bought baby spinach leaves were weighted 25 g in sterile sampling bag before inoculated with *E. coli* BL21 to achieve the final concentration of 10^2^ CFU/g. The bags were tightly sealed and kept at 4°C for 24 h. Then 225 ml of TSB was added to 25 g inoculated spinach in the sampling bags.

### Enrichment and Bacteriophage Infection

The enrichment of all samples was carried out at 37°C with constant shaking at 200 rpm for 5 h. For spinach leaf samples, 10 ml of the TSB was collected in a 50-ml centrifugal tube. Genetically engineered bacteriophage T7-ALP was added into 10 ml of the enriched samples with a concentration of 10^6^ PFU/ml. For liquid samples, the infection time was fixed at 30 min at 37°C with constant shaking at 200 rpm. For 25-g spinach leaf samples, the infection time was fixed at 2 h at 37°C with constant shaking at 200 RPM. Negative controls of each experiment were *E. coli* inoculated samples without bacteriophage infection and samples without bacteria inoculation with bacteriophage added.

### Colorimetric Assay of Alkaline Phosphatase Activity Using NBT/BCIP

After phage infection, 10 ml of TSB and coconut water samples, and 2 ml of spinach leaf samples were filtered through a 0.22-micron white polycarbonate membrane discs with a 19-mm diameter (Nucleopore Polycarbonate, Whatman) using a vacuum filtering system in order to capture infected bacterial cells harboring bacteriophage-induced alkaline phosphatase. After the filters were completely dry, 20 μl of 1-Step™ NBT/BCIP substrate solution (Thermo Scientific, Rockford, IL, United States) was spotted on a petri dish. The filter was then transferred directly onto the NBT/BCIP drop with topside down. The visible color change due to the formation of black-purple precipitated product was observed and compared with those of negative controls and tryptic soy broth without bacteria and bacteriophage. The Hunter’s color values (L*, a*, and b*) of the filters were measured at five locations of each filter using the ColorFlex EZ Spectrophotometer (Hunter Lab, Reston, VA, United States). The delta E (dE) value was calculated using Equation 1.

(1) $$\Delta E_{ab}^{*}=\sqrt{{\left(L_{2}^{*}-L_{1}^{*}\right)}^{2}+{\left(a_{2}^{*}-a_{1}^{*}\right)}^{2}+{\left(b_{2}^{*}-b_{1}^{*}\right)}^{2}}$$

### Colorimetric Assay of Alkaline Phosphatase Activity Using pNPP

After bacteriophage infection, 10 ml of all samples were centrifuged at 4025 ×*g* for 10 min at room temperature. The liquid media were discarded and the precipitated cells were re-suspended with 50 μl of sterile PBS. Amplite™ Colorimetric Alkaline Phosphatase Assay Kit (AAT Bioquest, Sunnyvale, CA) was used to perform the assay. After adding the substrate, the absorbance at 400 nm was measured every 10 min for 4 h using a SpectraMax 340 spectrophotometric plate reader (Molecular Devices, Sunnyvale, CA, United States). The experiments were performed in parallel with the blank, which was TSB without *E. coli* and bacteriophage inoculation and negative control of *E. coli* without bacteriophage inoculation.

### Statistical Analysis

All experiments were repeated three times. Color value was measured at five random positions on the filter, and the mean and standard deviation values were calculated within the samples. The Tukey’s HSD test were used to determine significant differences (*α* = 0.05) between mean values. All experimental data were analyzed using the R software.

## Results

### Colorimetric Detection of *E. coli* Using Engineered Bacteriophage T7-ALP and Alkaline Phosphatase Assay

**Figure 1:**
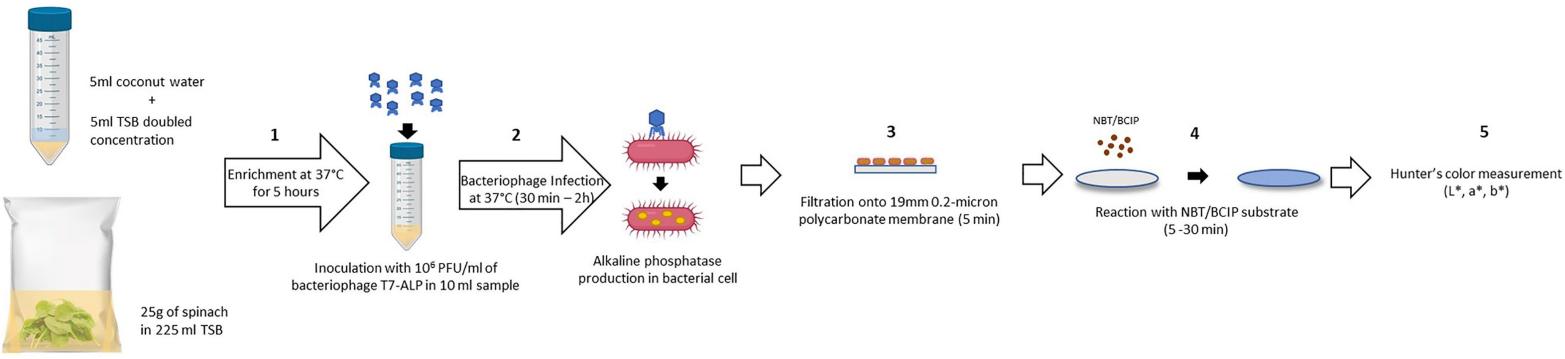
Schematic diagram of the detection protocol based on alkaline phosphatase activity assay using colorimetric alkaline phosphatase substrates: NBT/BCIP. (1) Enrichment of bacteria in spinach leaves without separation step (5 h). (2) Adding bacteriophage T7-ALP 10^6^ PFU/ml for the infection of *E. coli* and alkaline phosphatase expression (30 min – 2 h). (3) Filtration to harvest the infected *E. coli* cells (5 min). (4) Adding NBT/BCIP substrate for enzymatic reaction and forming of precipitated products (5–30 min). (5) Color measurement using colorimeter.

The schematic diagram (Figure 1) depicts the detection procedure developed in this study. One of the most challenging aspects in the detection of bacteria from a complex food system is the presence of food components and non-pathogenic microbes associated with the food. These elements can influence both the sensitivity and accuracy of the detection method. Developing a general protocol to separate and purify target cells from different food matrices is difficult as food matrices vary significantly in composition and structure (Frederick et al., 2013). In order to overcome these constraints, the bacteriophage-based detection method developed in this study requires no complicated sample preparation and bacterial separation or concentration steps. The food samples, coconut water, and baby spinach leaves were simply mixed with the TSB before enrichment and used for the detection of bacteria using a simple colorimetric approach. The steps for sample preparation are illustrated in Figure 1. The steps include enrichment of bacteria (step 1), incubation with T7-ALP (step 2), filtration (step 3), and incubation with ALP substrate to form colorimetric precipitate (step 4). The details of these steps are described in the Material and Methods section. Among these steps, the incubation and infection time was a critical factor in this assay, as our goal was to overexpress and entrap the expressed enzyme inside the bacterial cells, thus the infection time should be long enough for the expression of the enzyme but not too long to have complete lysis of the bacterial cells and release of the enzyme. This approach helps to localize the colorimetric signal on a filter and increase sensitivity of the detection. According to a previous study, after 30 min of co-incubation with bacteriophage T7-ALP, *E. coli* BL21 cells were infected and alkaline phosphatase was produced while the majority of the cells were still intact (Wisuthiphaet et al., 2019). By filtration (Figure 1; step 3), bacterial cells with alkaline phosphatase were captured on the filter and the colorimetric signal was concentrated on the filter membrane for visual detection.

Alkaline phosphatase activity was analyzed with the chromogenic substrate 5-bromo-4-chloro-3-indolyl phosphate (BCIP) that forms a dark purple precipitated produce (Figure 1; step 4). The color development was enhanced by nitro blue tetrazolium (NBT) yielding an insoluble black-purple precipitate as shown in the following reaction Equation 2 (Jékely and Arendt, 2007; Kundu, 2014).


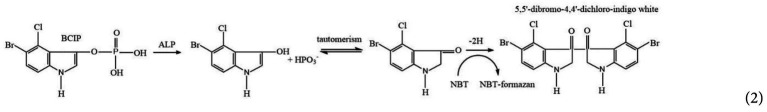


This enzymatic reaction took place on the filter. Therefore, the change in filter color to dark purple indicates the presence of the target bacteria. Color formation was observed visually as well as using a colorimeter (Figure 1; step 5). The detection method using a precipitated colorimetric substrate was compared to the soluble colorimetric alkaline phosphatase substrate, pNPP. In this method, infected cells with alkaline phosphatase were harvested by centrifugation before re-suspension in sterile PBS prior to the enzymatic reaction. Colorless pNPP was hydrolyzed to yellow p-nitro-phenol (pNP) in the presence of alkaline phosphatase and was quantified at 400 nm (Jackson et al., 2016).

### Detection of Bacteria Using Bacteriophage T7-ALP and Alkaline Phosphatase Substrate NBT/BCIP

**Figure 2:**
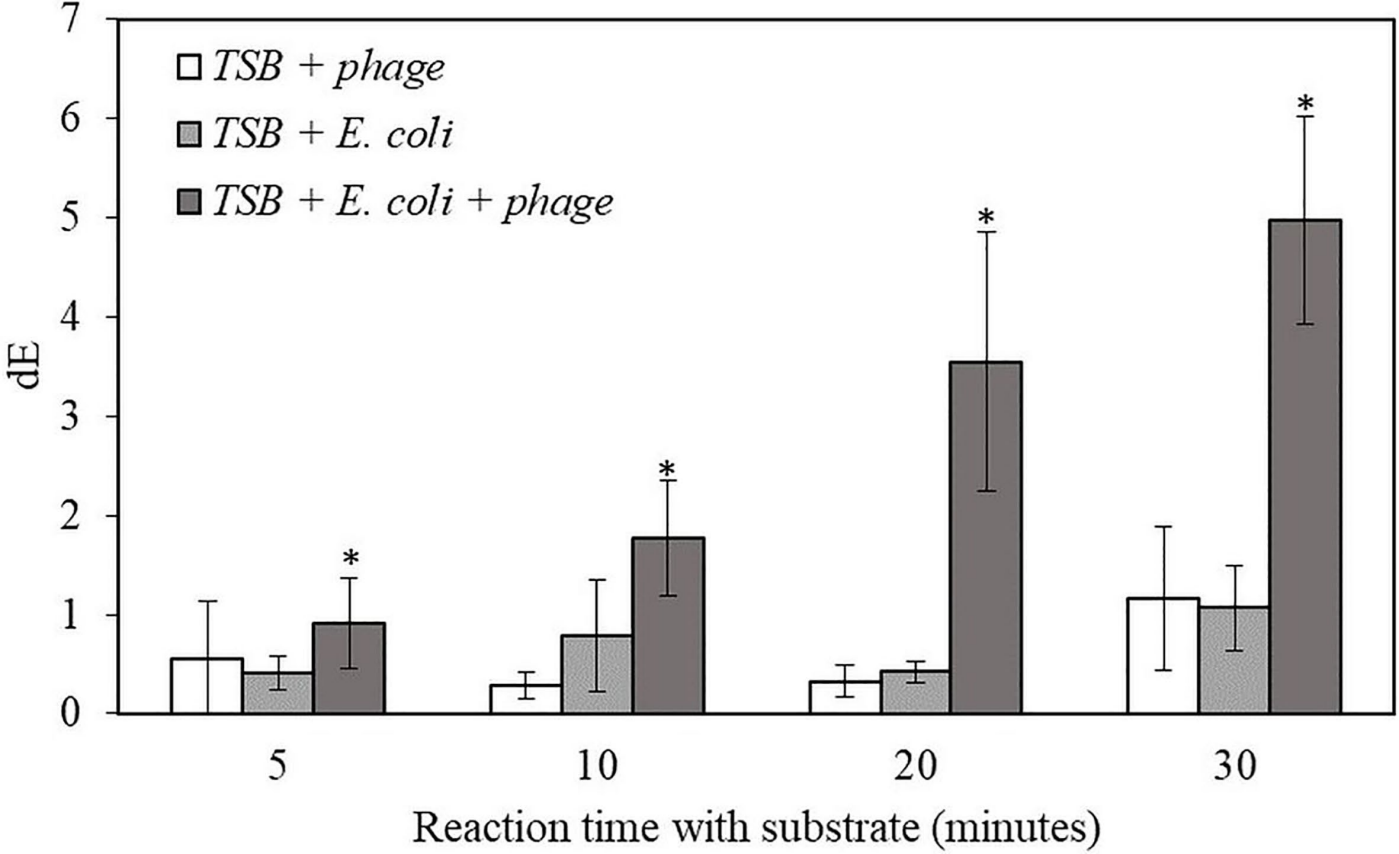
The dE value of the filter with bacteriophage T7-ALP in TSB, 5-h enriched 10 CFU/mL *E. coli* BL21 and 30 min with and without infection with bacteriophage T7-ALP in TSB after enzymatic reaction with NBT/BCIP for 5, 10, 20, and 30 min. Treatments with “*” are significantly different (*p* < 0.05). Error bars indicate ±standard deviation of means.

For the proof-of-concept demonstration, the detection of bacteria was first investigated in TSB, which is the media for supporting bacterial growth and bacteriophage infection. Table 1 shows the filters after adding NBT/BCIP substrate and incubating the filter at room temperature for 0, 5, 10, 20, and 30 min. For the samples with bacteriophage infection, after 10 min of incubation with the substrate, a visible dark purple color was observed and the color intensity increased with an increase in incubation time. In contrast, the negative control sample, i.e., filtered bacteria without bacteriophage infection showed no color change after 30 min of incubation with the substrate. The negative control of TSB with bacteriophage but without bacteria also showed no visible color change. In order to quantify the color change, the filters were also characterized using the Hunter’s color scale measurements using a colorimeter. In this measurement, the L*, a*, and b* color values were measured at random five locations of the filter and the dE values were calculated using the Equation 1. The dE value of the samples with T7-ALP infected bacteria and the negative control of bacteria without bacteriophage infection are shown in the Figure 2. After 5 min with the substrate, the dE value of the sample with T7-ALP infected bacteria is significantly higher than those of the controls and the dE value increases dramatically with an extended reaction time. On the other hand, the negative controls showed no significant change in the dE value after 20 min of incubation with the substrate.

**Table 1:** Filters with 5-h enriched 10 CFU/ml *Escherichia coli* BL21 and 30 min with and without infection with bacteriophage T7-ALP in TSB and bacteriophage T7-ALP in TSB after enzymatic reaction with NBT/BCIP for 5, 10, 20, and 30 min.

Reaction condition		Reaction time with substrate (min)				
*Escherichia coli* inoculation	T7 infection	0	5	10	20	30
+	+	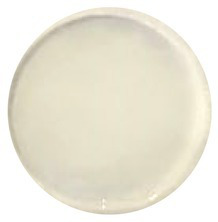	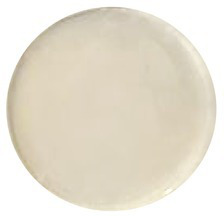	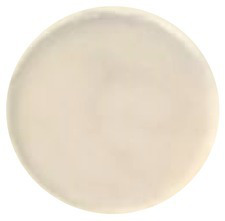	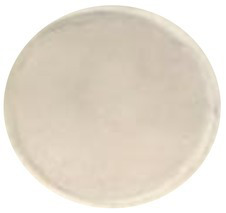	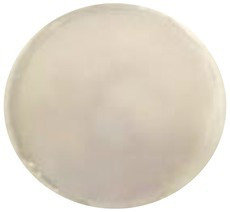
+	–	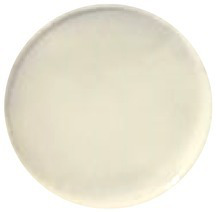	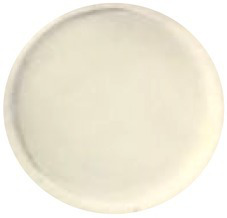	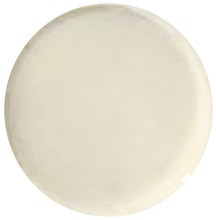	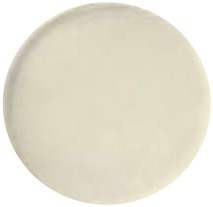	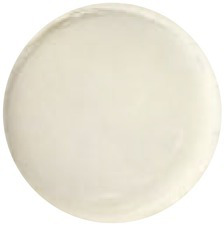
–	+	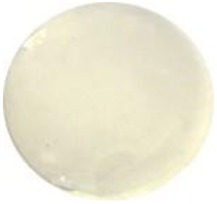	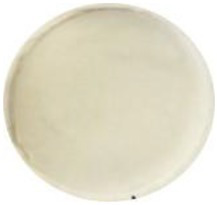	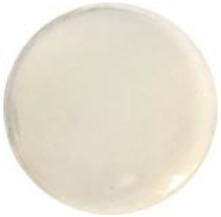	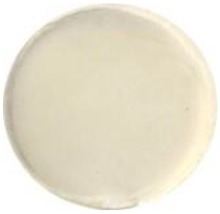	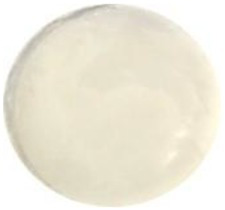

The results indicate that, after 30 min of infection, the *E. coli* cells were still intact and the alkaline phosphatase induced by T7-ALP infection was produced inside the cells, which were captured on the filter after filtration. The results of the negative control sample, i.e., bacterial cells without T7-ALP infection, demonstrate a lack of significant endogenous alkaline phosphatase activity in the bacteria since this enzyme is usually produced by the bacteria only during phosphate starvation (Rani et al., 2012). When the bacteria are enriched in TSB, a nutrient rich environment for bacterial growth, endogenous bacterial alkaline phosphatase enzyme is not highly expressed in bacterial cells. Overall, these results demonstrate that using a combination of engineered bacteriophage T7-ALP and colorimetric substrate NBT/BCIP, *E. coli* cells can be specifically detected at the initial inoculation levels of 10 CFU/ml within 6 h using a simple visual analysis or quantitative color imaging.

In order to validate this method using complex food materials, the detection of target bacteria in coconut water and baby spinach leaves were evaluated. Coconut water was selected as it represents a beverage product with sugars, fatty acids, and amino acids (Prades et al., 2012). Before bacterial inoculation, coconut water was mixed with a double concentrated TSB in order to provide nutrients to support bacterial growth. The samples were then inoculated with *E. coli* BL21 10 CFU/ml and incubated at 37°C for enrichment for 5 h followed by infecting the enriched sample with the T7-ALP bacteriophage for 30 min and the detection of the alkaline phosphatase activity after filtration of the coconut water sample. As shown in Table 2, after adding NBT/BCIP substrate for 10 min, the color change to dark purple color was observed and the color intensity increased with extended reaction time while the negative controls showed no color change after 30 min with the substrate.

**Table 2:** Filters with 5-h enriched 10 CFU/ml *E. coli* BL21 and 30 min with and without infection with bacteriophage T7-ALP in TSB-coconut water mixture and bacteriophage T7-ALP in TSB-coconut water mixture after enzymatic reaction with NBT/BCIP for 5, 10, 20, and 30 min.

Reaction condition		Reaction time with substrate (min)				
*Escherichia coli* inoculation	T7 infection	0	5	10	20	30
+	+	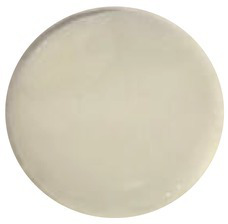	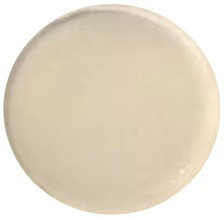	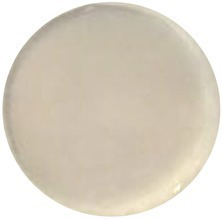	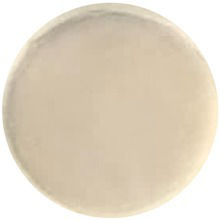	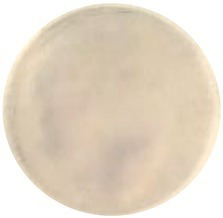
+	–	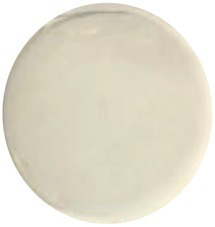	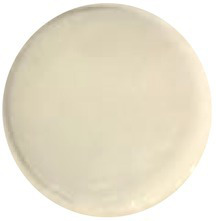	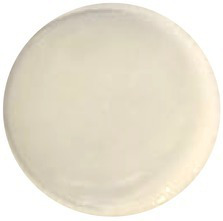	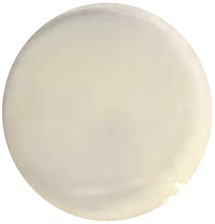	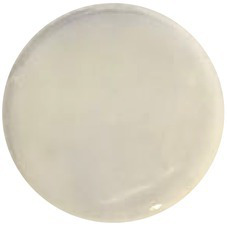
–	+	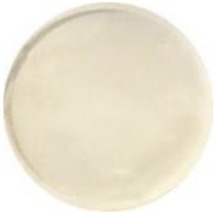	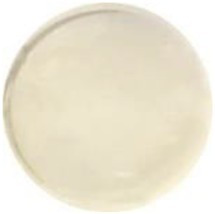	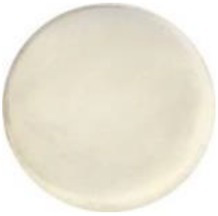	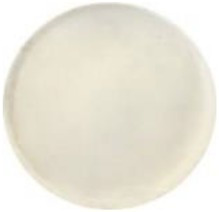	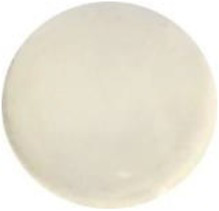

**Figure 3:**
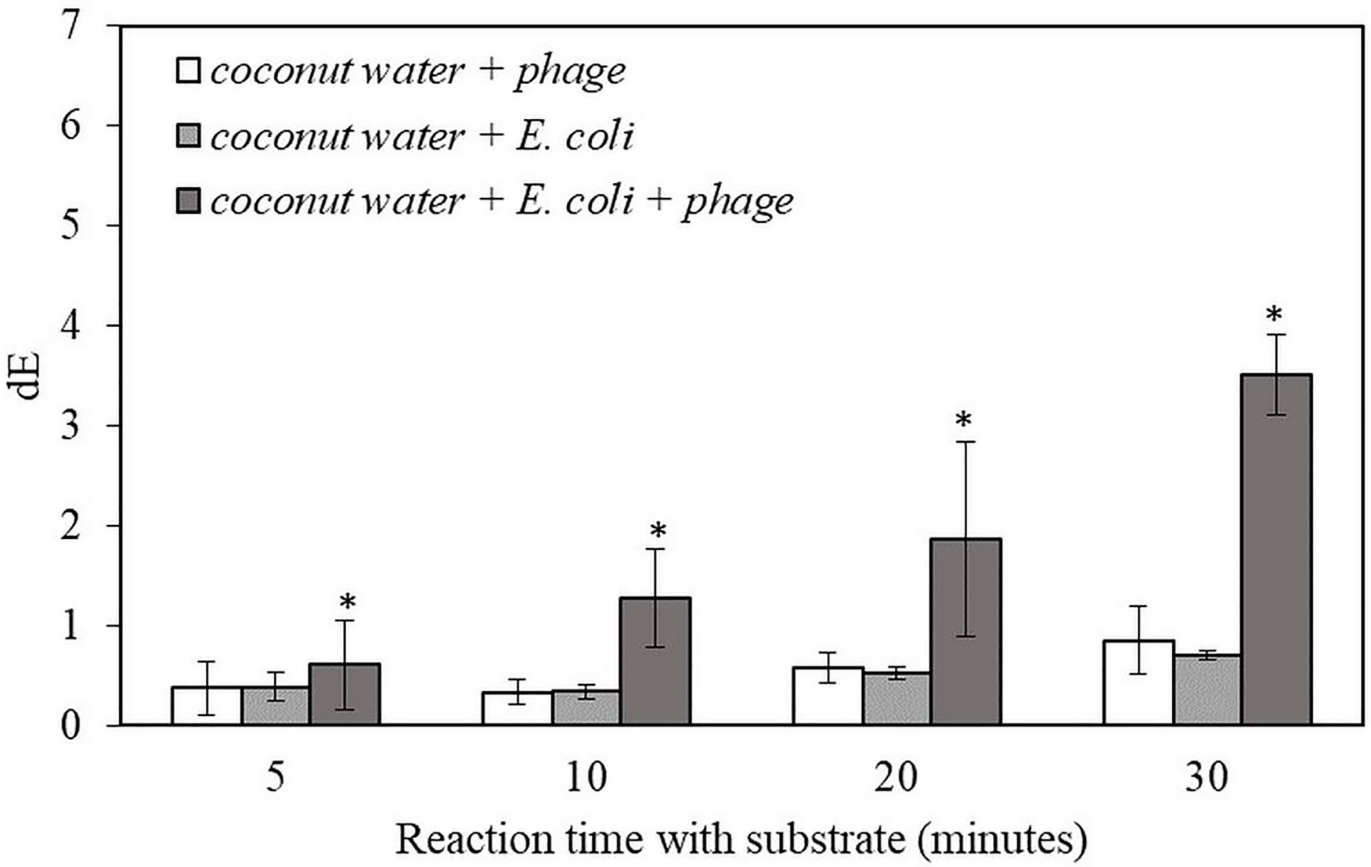
The dE value of the filter with bacteriophage T7-ALP in TSB-coconut water mixture, 5-h enriched 10 CFU/ml *E. coli* BL21 and 30 min with and without infection with bacteriophage T7-ALP in TSB after enzymatic reaction with NBT/BCIP for 5, 10, 20, and 30 min. Treatments with “*” are significantly different (*p* < 0.05). Error bars indicate ±standard deviation of means.

The result indicates that this detection method can be applied to detect 10 CFU/ml of the target bacteria in coconut water samples within 6 h. The samples with infected *E. coli* gave significantly higher dE value compared to negative controls. The dE value increased with longer reaction time while there was no increase in dE value for the negative controls (Figure 3). The results for the detection of bacteria inoculated in coconut water were similar to those of the detection of bacteria inoculated in pure TSB (Figure 2.). However, the dE values of the bacteria inoculated in coconut water samples were lower than those of bacteria inoculated in TSB, indicating reduced alkaline phosphatase expression or activity in coconut water. Therefore, the composition of coconut water may have an influence on the signal from alkaline phosphatase enzymatic reaction and the formation of the precipitated colorimetric product.

Fresh produce is one of the food samples that is considered as challenging for bacterial detection since the plant samples contain solid particles, pigments, and diverse microflora. In order to validate this detection method, baby spinach was selected to represent leafy greens. The sample size of baby spinach leaves was 25 g, which represent a more realistic sample size used in the industry. After inoculation with *E. coli* BL21, the sampling bags containing inoculated leaves were sealed and stored at 4°C for 24 h to simulated a scenario in the industry, where the harvested spinach leaves are stored at refrigerated temperature prior to washing and packaging. After 5 h of enrichment, spinach samples were then incubated with the bacteriophage T7-ALP for 2 h. The extended infection time was selected to reduce the influence of non-target microbes and the plant exudate from the spinach leaves. The release of plant exudate from the wound and cut of spinach leaves results in a green color that may interfere with the color change measurement induced by the overexpression of alkaline phosphatase and its colorimetric substrate. Moreover, an increase in the level of non-target bacteria may slow the growth of *E. coli* during enrichment and may physically obstruct the binding of bacteriophage and the target bacteria. Increase in infection time to 2 h allows an additional growth of *E. coli* during incubation with bacteriophages and increase the infection efficiency. Detection of *E. coli* in small scale of 1-gram spinach leaves was also conducted. With less interference from the plant exudate, the optimal coincubation time was 30 min which the result is reported in the supplementary material.

**Figure 4:**
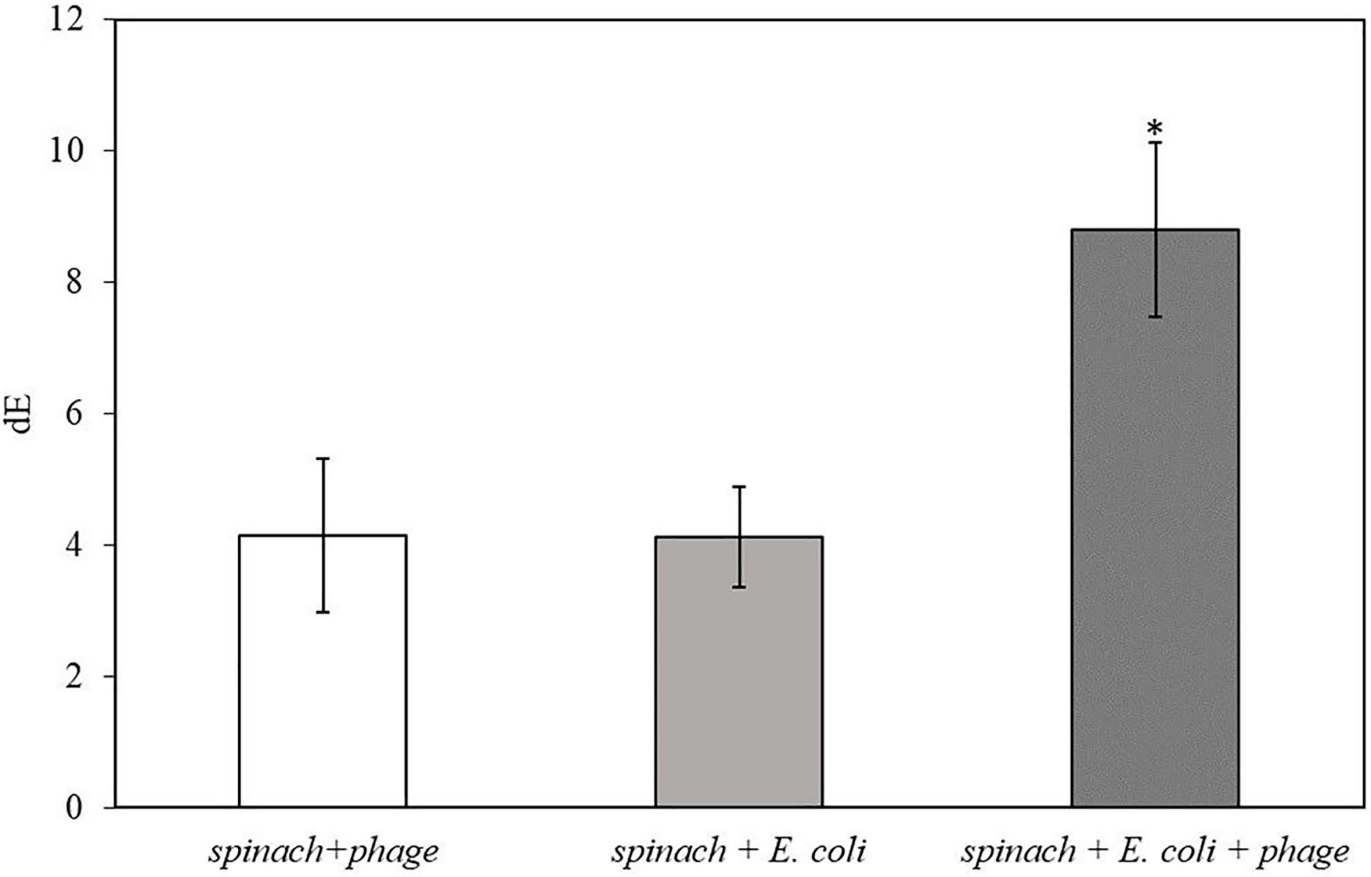
The dE of the filter with 5 h-enriched 10 CFU/ml *E. coli* BL21 and 2 h infection with bacteriophage T7-ALP in 225 ml TSB with 25 g spinach leaves after enzymatic reaction with NBT/BCIP for 30 min. Treatments with “*” are significantly different (*p* < 0.05). Error bars indicate ±standard deviation of means.

After 2 h of infection with bacteriophage, 2 ml of enriched TSB was filtered through a 0.2-micron polycarbonate filter. Since the spinach samples contain solid plant particles and also native microflora from plant tissue that would clog the pores of the filter, the volume of sample subjected to filtration was limited to 2 ml. As shown in Table 3, the filters after filtration appeared slightly green due to the presence of released plant pigments. After 30 min of enzymatic reaction with the NBT/BCIP substrate, the filter appeared darker and the dE value of inoculated spinach samples with bacteriophage infection on a filter was significantly higher than the negative controls (Figure 4.). The results demonstrate that this detection method can be applied to detect bacteria using a realistic spinach sample size without isolating bacteria from the inoculated plant samples.

**Table 3:** Filters with 5-h enriched 10 CFU/ml *E. coli* BL21 and 30 min with and without infection with bacteriophage T7-ALP in TSB-spinach and bacteriophage T7-ALP in TSB-spinach after enzymatic reaction with NBT/BCIP for 0 and 30 min.

Reaction condition		Reaction time with substrate (min)	
*Escherichia coli* inoculation	T7 infection	0	30
+	+	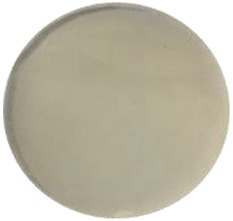	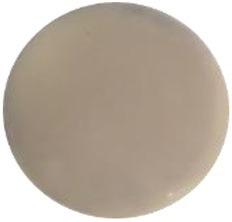
+	–	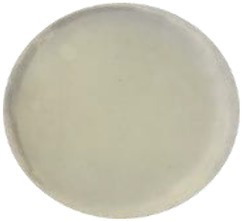	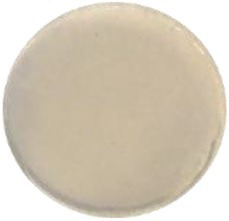
–	+	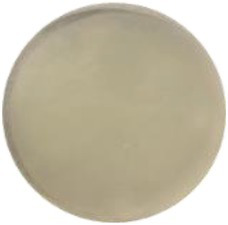	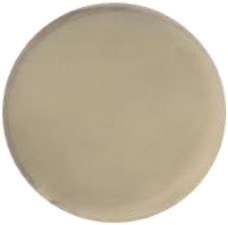

### Detection of Bacteria Using Bacteriophage T7-ALP and Alkaline Phosphatase Substrate pNPP

To detect the expression of alkaline phosphatase using colorimetric assays, often substrates, such as pNPP, that generate a soluble colorimetric signal are used. The advantage of the homogeneous assay is the ease of detection using a simple UV–Vis measurement. In this study, one of our objectives was to compare the sensitivity of colorimetric detection of target bacteria using both insoluble and soluble enzymatic substrates. For this comparison, the experimental conditions for the enrichment and infection steps were maintained the same as in the case of when using the substrate that yields precipitated products, NBT/BCIP. The only difference was that after initial infection, the cells were harvested using a centrifuge and then the isolated cell pellet was re-suspended in PBS and incubated with the pNPP substrate as described in the Materials and Methods section. The absorbance of the resulting solution was measured at 400 nm using a UV–Vis spectrophotometer.

**Figure 5:**
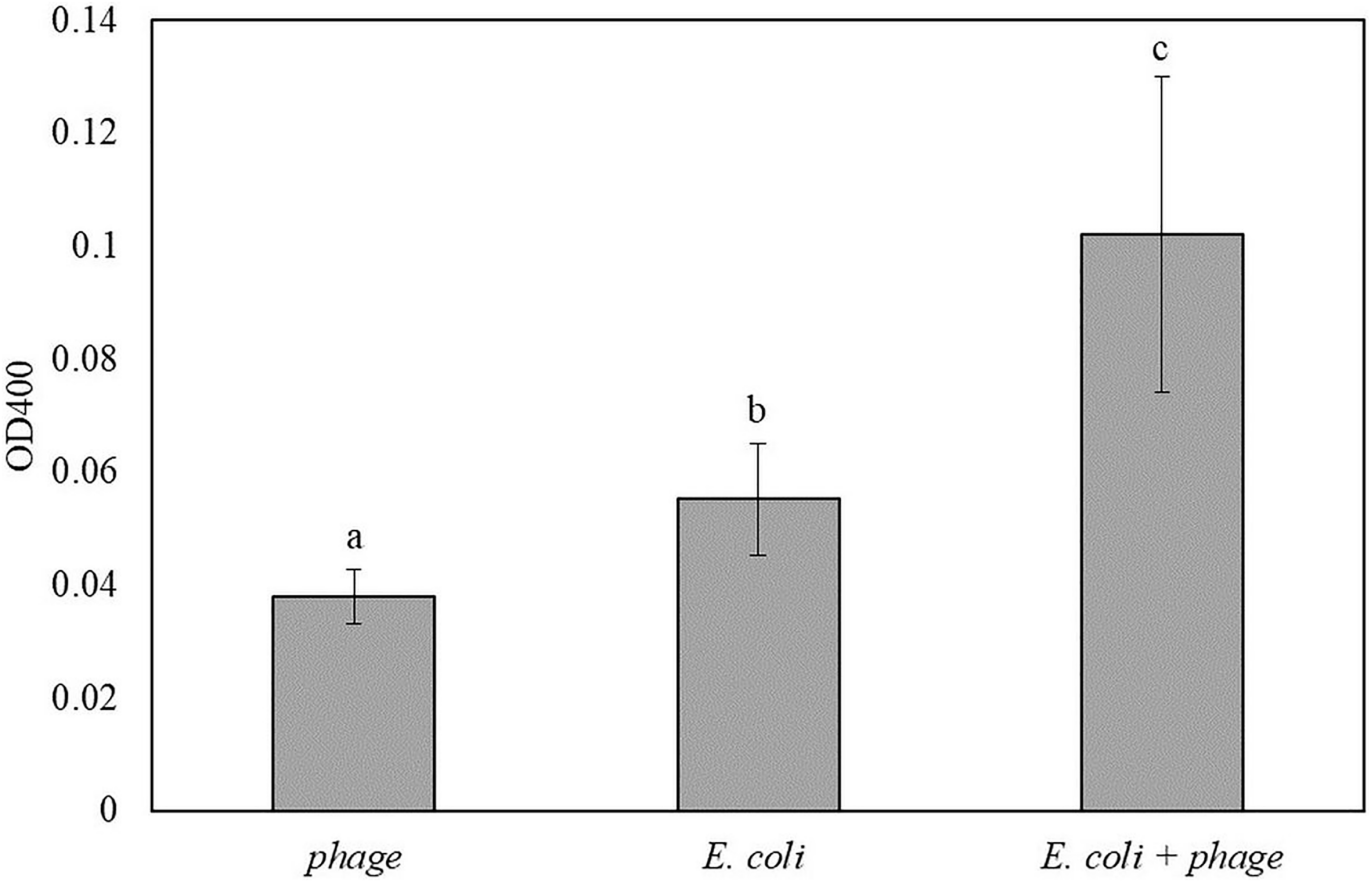
The OD400 of 5 h-enriched 10 CFU/ml *E. coli* BL21 and 30 min infection with bacteriophage T7-ALP in TSB after enzymatic reaction with *p*NPP for 2 h. Treatments with different letters are significantly different (*p* < 0.05) error bars indicate ±standard deviation of means.

Figure 5 shows the results for the detection of *E. coli* in TSB that was enriched for 5 h and infected with bacteriophage T7-ALP for 30 min. After 2 h of incubation with pNPP substrate, *E. coli* infected with bacteriophage had significantly higher absorbance compared to negative controls. For the soluble substrate assay, bacteria are incubated in PBS for 2 h and this incubation of bacteria in a low nutrient environment can increase the endogenous expression of alkaline phosphatase compared to bacteria incubated with TSB. Increase in an endogenous expression of alkaline phosphatase can reduce the sensitivity of the detection using the soluble substrate.

**Figure 6:**
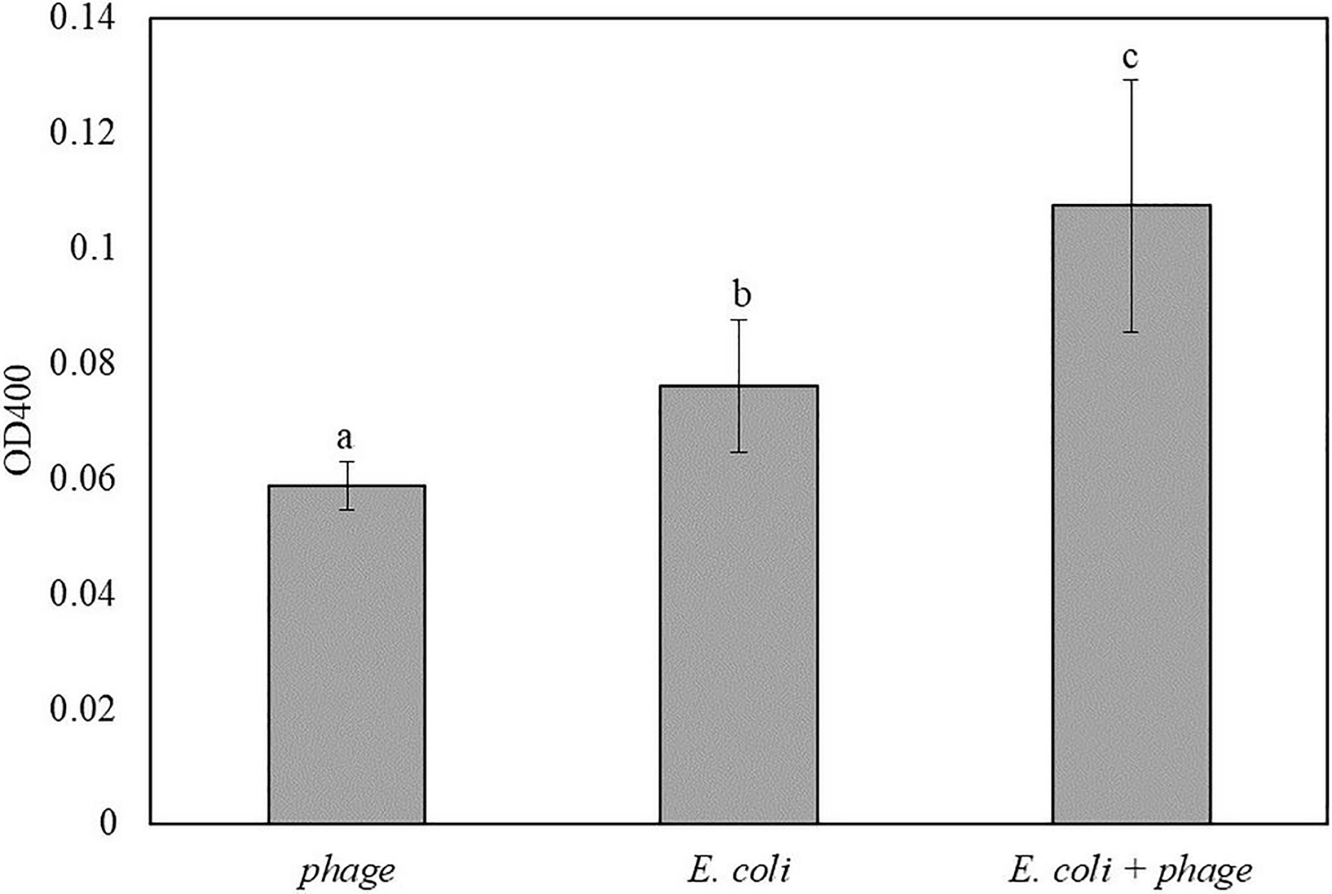
The OD400 of 5 h-enriched 10 CFU/ml *E. coli* BL21 and 30 min infection with bacteriophage T7-ALP in coconut water-TSB after enzymatic reaction with *p*NPP for 2 h. Treatments with different letters are significantly different (*p* < 0.05) error bars indicate ±standard deviation of means.

Similar to the results in Figure 5, coconut water was used as a model liquid food system for the detection of *E. coli*. The mixture of 1:1 coconut water and double-concentrated TSB inoculated with 10 CFU/ml of *E. coli* was enriched for 5 h followed by 30 min of infection with bacteriophage T7-ALP. Then the bacterial cells were incubated with the soluble substrate for 2 h. The inoculated samples with bacteriophage infection had the highest OD400 followed by coconut water with *E. coli* without bacteriophage infection and coconut water and bacteriophage without *E. coli*, respectively (Figure 6). The trend was similar to those of bacteria in TSB alone.

This method using soluble substrate was not applicable for the detection of *E. coli* in 25 g of spinach samples and larger volume of media since enrichment steps results in the growth of other local microbes, and the centrifugation also harvested plant particles along with microbial cells, resulting in high turbidity after re-suspension, which interfered with the absorbance measurement.

## Discussion

Detection of specific bacteria in food matrices is a challenging task. This challenge results because of low counts of target bacteria in food systems and also the interference due to the food matrix and other microbes. To address these challenges various processes have been designed to isolate, concentrate, and separate target bacteria from the food matrix using both physical and biochemical methods. Physical methods, such as filtration and centrifugation, can improve the detection sensitivity but their drawback is that the solid food debris will also be concentrated along with bacteria (Benoit and Donahue, 2003) and can further limit detection sensitivity. Immunomagnetic separation has been used coupled with several bacterial detection methods in order to specifically separate target bacteria from food debris and other endogenous microbes. The significant challenge results due to the binding affinity and specificity of antibodies that can be reduced by food components due to the diversity of mechanisms (Van Aken and Lin, 2011). Moreover, an attachment of bacteria to food surface can significantly limit the overall capture efficiency using both physical and biochemical methods. In addition to these constraints, sample preparation for the detection of bacteria in food materials is often one of the key labor and resource intensive steps with a series of manual handling steps to prepare samples for enrichment and detection. These handling practices can lead to contaminations of the samples especially when multiple samples are being processed simultaneously (Benoit and Donahue, 2003). To overcome these limitations, the detection methods developed in this study require no sample preparation steps to isolate bacteria from diverse food matrices. Coconut water and baby spinach leaf samples, representing a liquid and a fresh produce model system inoculated with bacteria were added directly to the bacterial enrichment media that can enrich a diversity of bacteria, thus providing a general framework for the detection of bacteria. Furthermore, the time required for bacterial enrichment step was comparable or less than the average enrichment time used prior to other detection methods, such as RT-PCR and other nucleic acid or ELISA based methods (De Boer and Beumer, 1999), and was achieved in less than a single work shift in the industry. In addition, unlike PCR or other nucleic acid-based methods, the detection approach developed in this study does not require isolation and purification of nucleic acids. Thus, overall, the simple workflow of the method described in the study significantly reduces the labor and resources typically required for the sample preparation and detection of bacteria. These simplifications combined with visual detection of bacteria using colorimetric analysis provide a simple yet effective approach to detect bacteria in food systems.

Colorimetric detection provides simple and low-cost operations since it does not require any advanced instruments, which allows portable and easy-to-use diagnostic. Prior studies have developed colorimetric assays for *E. coli* detection tested in culture media, drinking water, and liquid food matrices as summarized in Table 4.

**Table 4:** Summary of the previous studies on colorimetric bacteriophage-based detection of *E. coli*.

Sample matrices	Colorimetric detection approach	Detection limit	References
Drinking water	Chromogenic soluble substrate detection of β-galactosidase released after phage-induced lysis of *Escherichia coli*	10 CFU/ml with 6 h of pre-enrichment	Chen et al., 2015
Culture medium	Detection of alkaline phosphatase overexpression induced by engineered bacteriophage T7 using pNPP	10^4^ CFU/ml within 7.5 h	Alcaine et al., 2015
Culture medium	Detection of β-galactosidase induced by engineered T7 phage infection on *Escherichia coli*	10 CFU/ml within 7 h	Chen et al., 2017a
Drinking water, Skim milk, and Orange juice	Detection of β-galactosidase induced by freeze-dried engineered T7 phage infection on *Escherichia coli*	10^2^ CFU/ml within 7 h	Chen et al., 2017b
Culture medium 100 ml	Detection of alkaline phosphatase overexpression induced by engineered bacteriophage T7 infection on *Escherichia coli*	<10 CFU/ml within 8 h	Singh et al., 2019

To the best of our knowledge, most of these studies have focused on using soluble substrates whose chromatic products will be dissolved in the bulk solution. This limits the detection sensitivity compare to using substrates that yield localized enzymatic products inside the infected cells. Moreover, using soluble substrates requires the release of enzyme from cells to the aqueous phase, which may not be efficient as alkaline phosphatase have high molecular weight of 94 kDa. In our prior research, we have observed a significant retention of cellular content including DNA and cell membrane after bacteriophage lysis of cells (Yang et al., 2020). Thus, an extended time of incubation may result in more efficient release of enzymes from residual cellular content after lysis as well as multiple repeat cycles of phage infection and the expression of exogenous enzyme can increase the effective enzyme concentration.

In order to increase the sensitivity and simplicity of the detection, the substrate that forms colorimetric precipitated product after the enzymatic reaction, NBT/BCIP, were investigated in this study. This substrate has been used as the substrate for alkaline phosphatase in the *in-situ* hybridization for gene expression study (Jékely and Arendt, 2007; Trinh et al., 2007). The dark purple insoluble precipitated product is the result of the enzymatic reaction, and it can be visualized by the naked eye. The use of this substrate to detect bacterial alkaline phosphatase was supported by the study of Hinkley et al. (2018), which reported that BCIP substrate can be used to visualize bacterial colonies infected by engineered bacteriophage with alkaline phosphatase. This method allows quantitative analysis of bacteria within 10 h of operation (Hinkley et al., 2018). For the detection method developed in this study, the samples after bacteriophage infection were filtered to capture bacterial cells with alkaline phosphatase. Since the product of NBT/BCIP is water insoluble and will localize inside bacterial cells, the release of enzyme was not required. The enzymatic product can be concentrated simultaneously with the bacterial cells by filtration, which can improve the detection sensitivity. The substrate can be applied directly to the filter and the color formation can be visualized within 10 min without any instruments or imaging system. As bacteriophage T7 is highly specific to *E. coli* infection; therefore, other bacteria present in the sample will not be infected thereby there is no overexpression of alkaline phosphatase, which eliminates the cause of false-positive result. Prior to this study, the T7-ALP has been tested against with food and agriculture related bacterial strains, including *Pseudomonas fluorescens* and *Listeria innocua*, and the results indicated no significant increase of alkaline phosphatase after bacteriophage T7-ALP infection (Wisuthiphaet et al., 2019).

Compared to the previous colorimetric bacteriophage-based detections, the detection approach developed in this study offers a rapid detection method that can be applied to detect target bacteria in beverage and fresh produce samples with more realistic sample size. The results indicated the successful detection of bacteria 10 CFU/ml and 10^2^ CFU/g in coconut water and spinach leaves with 5-h enrichment. However, for the food samples containing solid particles and pigments like spinach, the filtration step could limit the detection efficiency. Due to the solid plant particles, the sample volume for filtration is limited, which affects the detection sensitivity. Moreover, the leaf pigments can interfere with the color readout. The higher sample volume results in the more food debris and local microbes, which leads to reduced filtration volume and detection sensitivity. Therefore, the reaction time was extended to 2 h to increase the alkaline phosphatase level to achieve the detection limit of 10^2^ CFU/g of bacteria. The detection using pNPP substrate is not applicable since the centrifugation step did sediment all the debris and local microbes that might outgrow the target bacteria causing high turbidity of the samples and prevent an accurate absorbance measurement.

Further refinement of the detection procedure is still needed in order to develop more efficient detection methods that can be applied to detect bacteria in complex food samples by focusing on eliminating the background noise from food particles and non-target microbes. Multistep filtration can be introduced to remove coarse suspended solids in the samples before harvesting the bacteriophage-infected bacteria. Selective media can be applied during the enrichment step to support the growth of the target bacteria and reduce the proportion of other non-target microbes. In addition, quantitative analysis of bacteria using this detection method can be further investigated as well as combining this detection approach with digital imaging and image processing also improve the detection specificity and sensitivity.

## Conclusion

This study demonstrates an isolation-free rapid bacterial detection strategy using engineered bacteriophage to induce alkaline phosphatase expression. The NBT/BCIP substrate of alkaline phosphatase allows the rapid colorimetric bacterial detection. The proposed method was validated to detect *E. coli* in complex food samples, coconut water, and baby spinach. The detection limit of 10 CFU/ml of bacteria in coconut water was achieved within 6 h of operation. For baby spinach leaves, 10^2^ CFU/g of bacteria can be detected with an extended detection time of 8 h. Further study may be conducted in order to address some of the drawbacks including limitations of filtering large volume samples with food particulate matter and analyzing large number of samples.

## Data Availability Statement

The original contributions presented in the study are included in the article/supplementary material, and further inquiries can be directed to the corresponding author.

## Author Contributions

NW designed the study and performed the experiments, analyzed the data, and wrote the manuscript. XY designed the study and performed the experiments. NN and GY conceived, designed, and supervised the study. All authors contributed to the article and approved the submitted version.

## Conflict of Interest

The authors declare that the research was conducted in the absence of any commercial or financial relationships that could be construed as a potential conflict of interest.

## Publisher’s Note

All claims expressed in this article are solely those of the authors and do not necessarily represent those of their affiliated organizations, or those of the publisher, the editors and the reviewers. Any product that may be evaluated in this article, or claim that may be made by its manufacturer, is not guaranteed or endorsed by the publisher.
