# Supplementary material for: Application of Engineered Bacteriophage T7 in the Detection of Bacteria in Food Matrices
Source: Front Microbiol. 2021 Aug 6;12:691003. doi: 10.3389/fmicb.2021.691003 (PMC8377434; doi:10.3389/fmicb.2021.691003)
Supplement: Supplementary file 1 [file Data_Sheet_1.DOCX]

Supplementary Material

# Detection of bacteria in 1g baby spinach using bacteriophage T7-ALP and alkaline phosphatase substrate NBT/BCIP

Baby spinach was weighted 1g before inoculation with *E. coli* then kept in sealed petri dish for 24 hours at 4°C in order to enhance the attachment of bacterial cells onto the leaves’ surfaces. The inoculated leaves were directly put into 10 ml TSB and incubated at 37 °C for 5 hours before adding bacteriophage T7-ALP for infection. After 30 minutes of infection, 2 ml of the sample was filtered through the 0.2-micron polycarbonate filter.

However, after adding NBT substrate, the color change can be visualized after 5 minutes of incubation with the substrate as shown in the Supplementary Table 1. To ensure that the spinach leaf itself doesn’t affect the color change during incubation, the non-inoculated spinach control was tested and the results showed a slight change to a darker color only after 20 minutes with the substrate. However, as shown in Supplementary Figure 1, there was a significant difference in dE value of the inoculated samples compared to the negative controls after 20 minutes of incubation with the substrate. The dE value of inoculated spinach with phage infection shows higher variation in signal. This variation was attributed due to the influence of non-homogenous plant particles deposited on the filter.

**Supplementary Table 1** Filter with bacteriophage T7-ALP infected 5-hour enriched 10 CFU/g *E. coli* BL21 in TSB-spinach after 0, 5, 10, 20, and 30 minutes of enzymatic reaction with alkaline phosphatase substrate, NBT/BCIP

|  | Reaction time with substrate (minutes) | | | | |
| --- | --- | --- | --- | --- | --- |
|  | 0 | 5 | 10 | 20 | 30 |
| With T7-ALP phage infection | **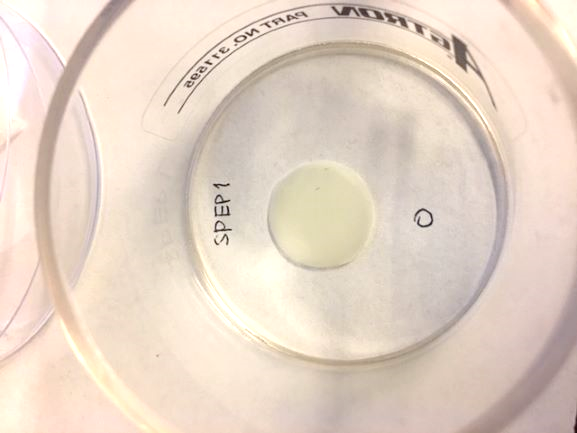** | **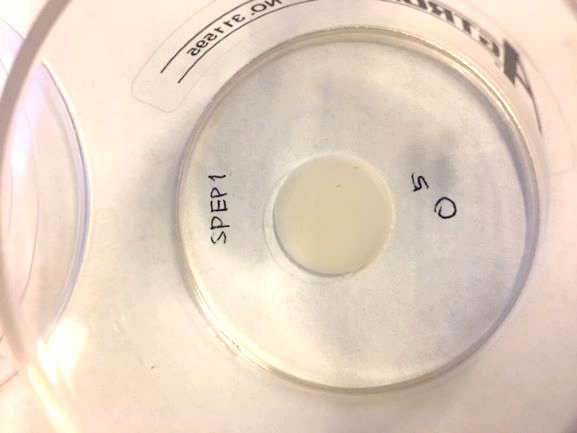** | **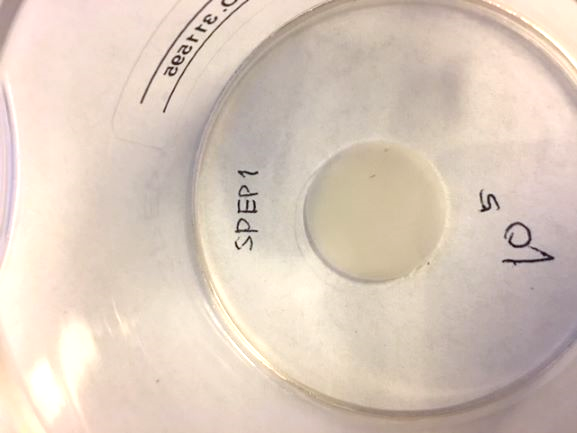** | **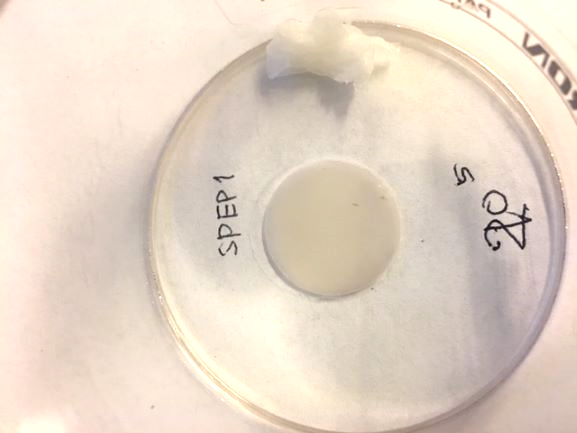** | **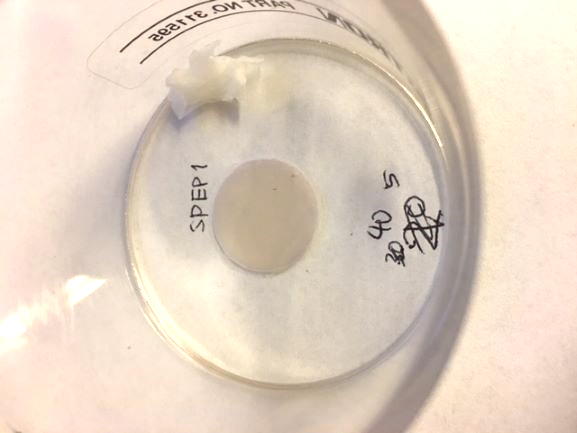** |
| No T7-ALP phage infection | **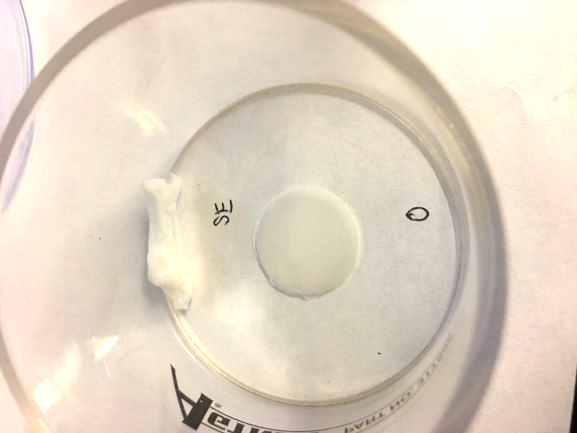** | **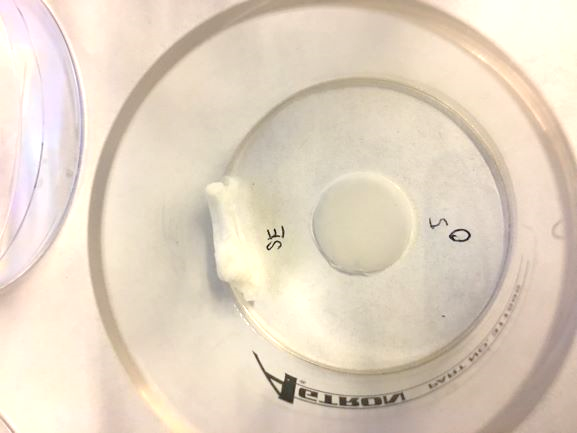** | **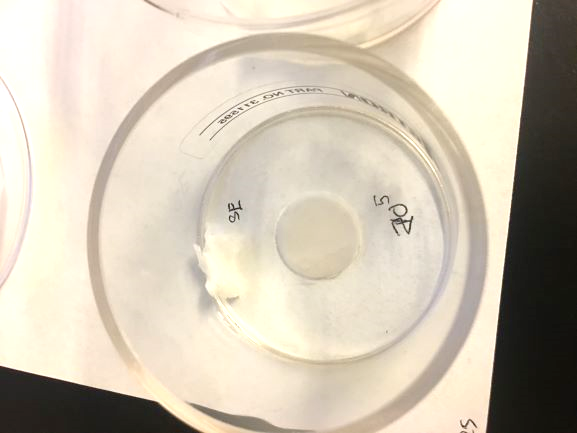** | **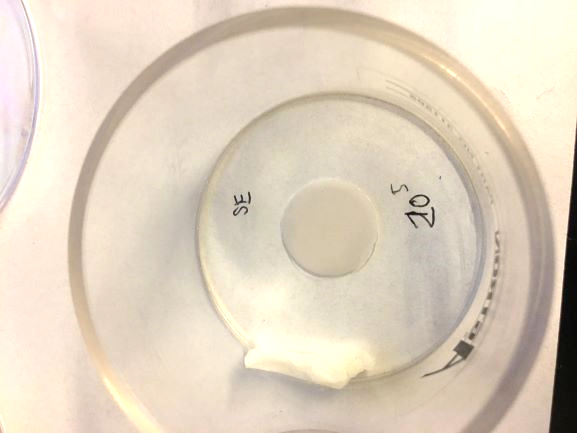** | **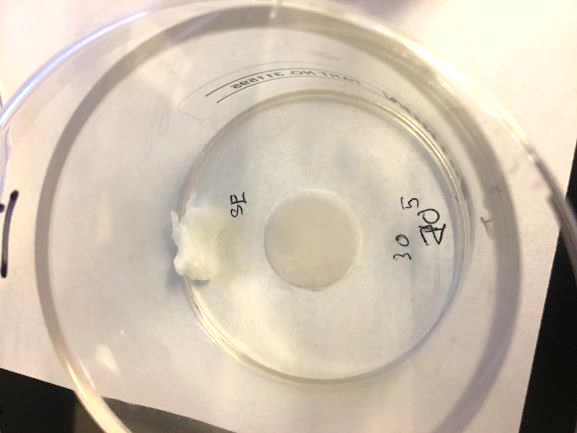** |


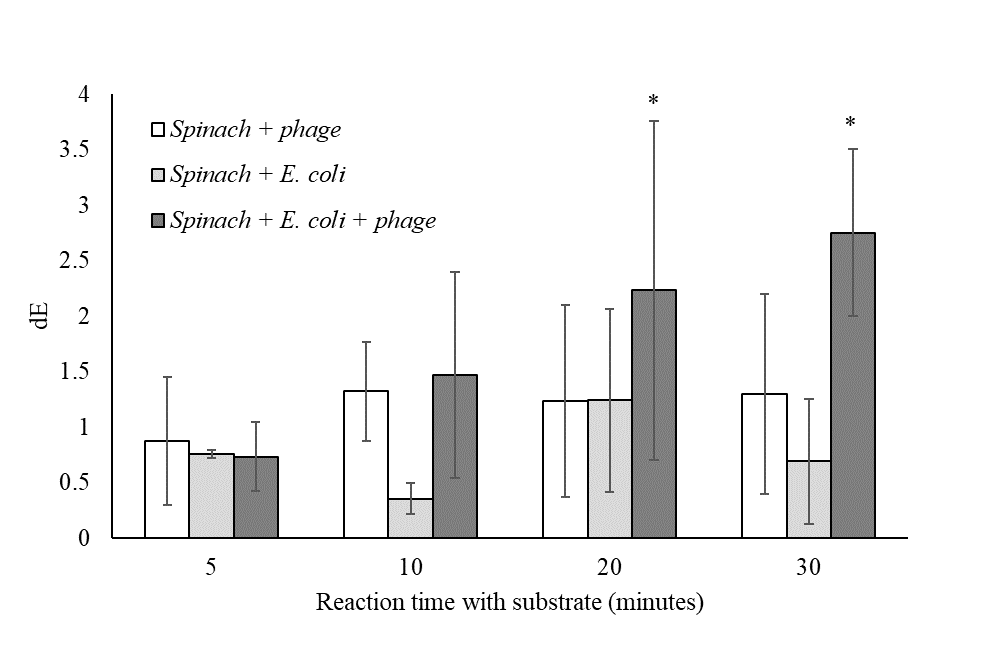


**Supplementary Figure 1.** The dE value of the filter with bacteriophage T7-ALP in TSB-spinach, 5-hours enriched 10 CFU/mL *E. coli* BL21 and 30 minutes with and without infection with bacteriophage T7-ALP in TSB after enzymatic reaction with NBT/BCIP for 0, 10, 20, and 30 minutes. Treatments with ‘*’ are significantly different (p < 0.05). Error bars indicate ±standard deviation of means.

# Detection of bacteria in 1g baby spinach using bacteriophage T7-ALP and alkaline phosphatase substrate pNPP

Supplementary Figure 2 shows the result when *E. coli* 10 CFU/ml was inoculated on the surface of 1 g of baby spinach leaves. With phage infection, the OD400 was significantly higher that the negative controls after 2 hours and 30 minutes of reaction with the substrate. Spinach with bacteriophage gave slightly higher OD400 than those of inoculated spinach without phage infection. This can be due to the fact that some local bacteria present on the leave can also be affected by the bacteriophage T7-ALP and the production of the enzyme was induced. The signal from spinach samples were higher than those of coconut water and TSB since there might be a noise background from alkaline phosphatase produced by other native microbes.


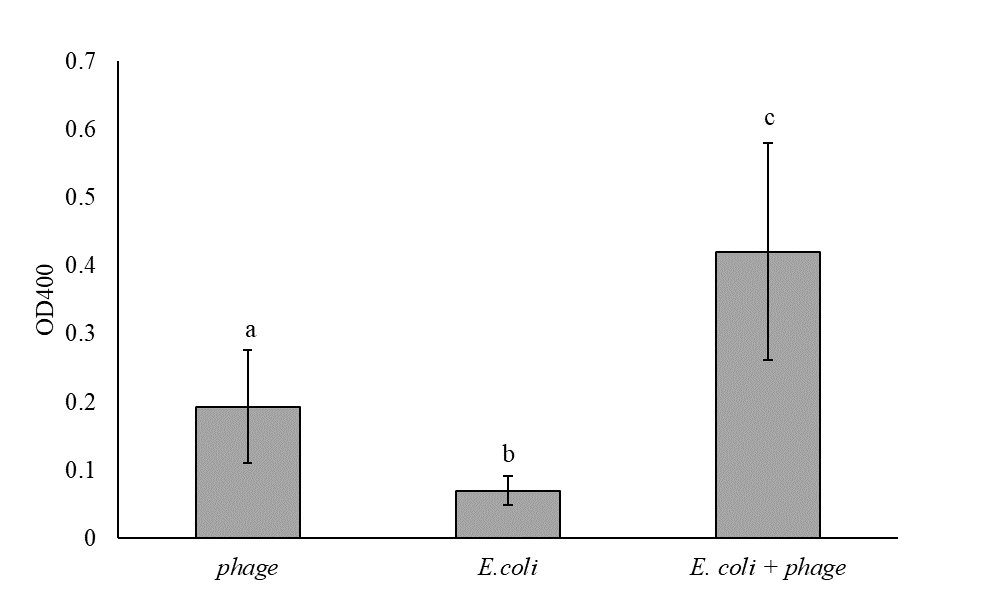


**Supplementary Figure 2.** The OD400 of 5 hours-enriched 10 CFU/mL *E. coli* BL21 and 30 minutes infection with bacteriophage T7-ALP in spinach after enzymatic reaction with pNPP for 2 hours. Treatments with different letters are significantly different (p < 0.05) error bars indicate ±standard deviation of means.
